# Supplementary material for: T-cell co-stimulation in combination with targeting FAK drives enhanced anti-tumor immunity
Source: eLife. 2020 Jan 21;9:e48092. doi: 10.7554/eLife.48092 (PMC6974352; doi:10.7554/eLife.48092)
Supplement: Supplementary file 1. [file elife-48092-supp1.pptx]

## Slide 1
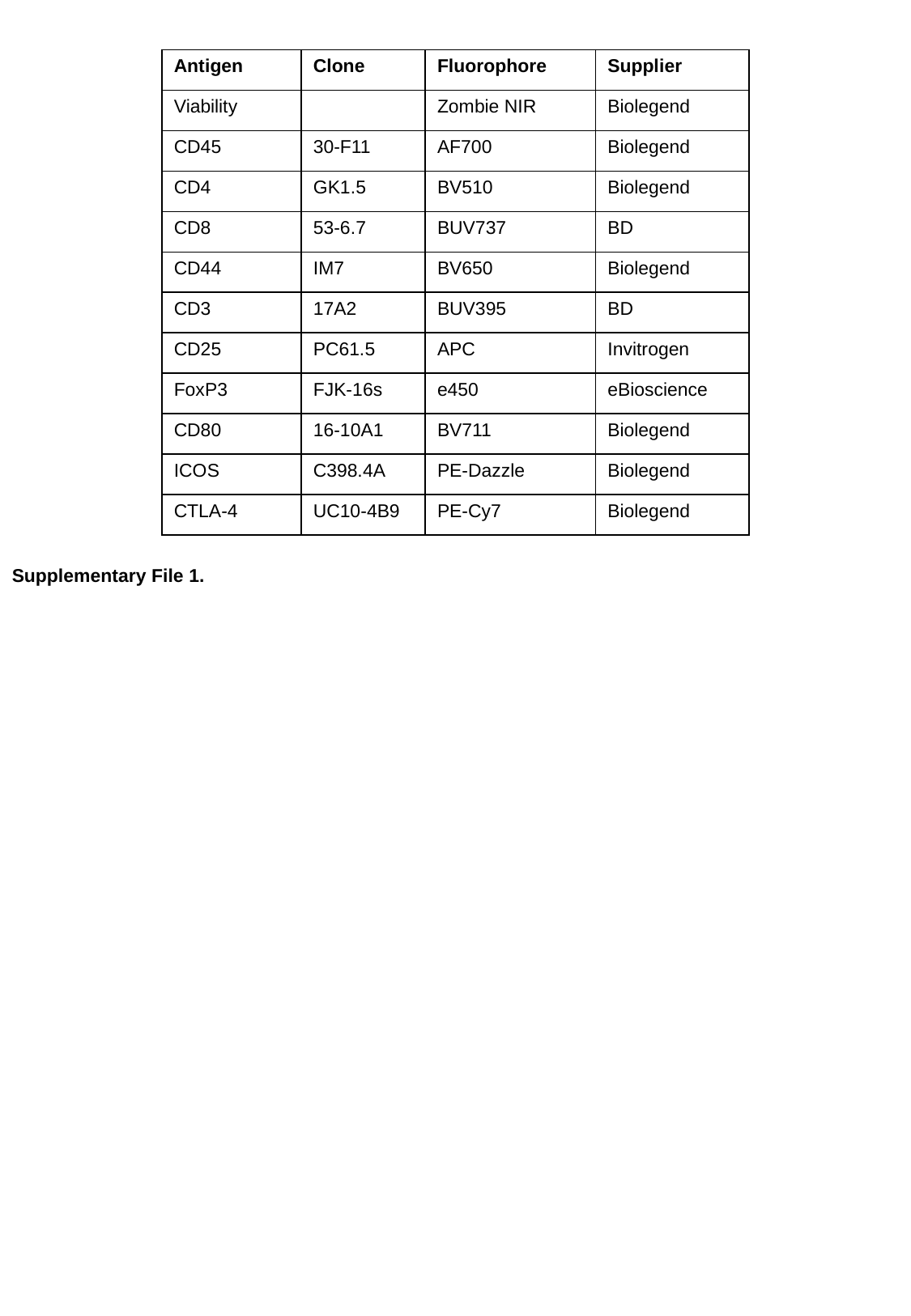

| Antigen | Clone | Fluorophore | Supplier |
| --- | --- | --- | --- |
| Viability | | Zombie NIR | Biolegend |
| CD45 | 30-F11 | AF700 | Biolegend |
| CD4 | GK1.5 | BV510 | Biolegend |
| CD8 | 53-6.7 | BUV737 | BD |
| CD44 | IM7 | BV650 | Biolegend |
| CD3 | 17A2 | BUV395 | BD |
| CD25 | PC61.5 | APC | Invitrogen |
| FoxP3 | FJK-16s | e450 | eBioscience |
| CD80 | 16-10A1 | BV711 | Biolegend |
| ICOS | C398.4A | PE-Dazzle | Biolegend |
| CTLA-4 | UC10-4B9 | PE-Cy7 | Biolegend |
Supplementary File 1.
