# Supplementary material for: T-cell co-stimulation in combination with targeting FAK drives enhanced anti-tumor immunity
Source: eLife. 2020 Jan 21;9:e48092. doi: 10.7554/eLife.48092 (PMC6974352; doi:10.7554/eLife.48092)
Supplement: Supplementary file 3. [file elife-48092-supp3.pptx]

## Slide 1
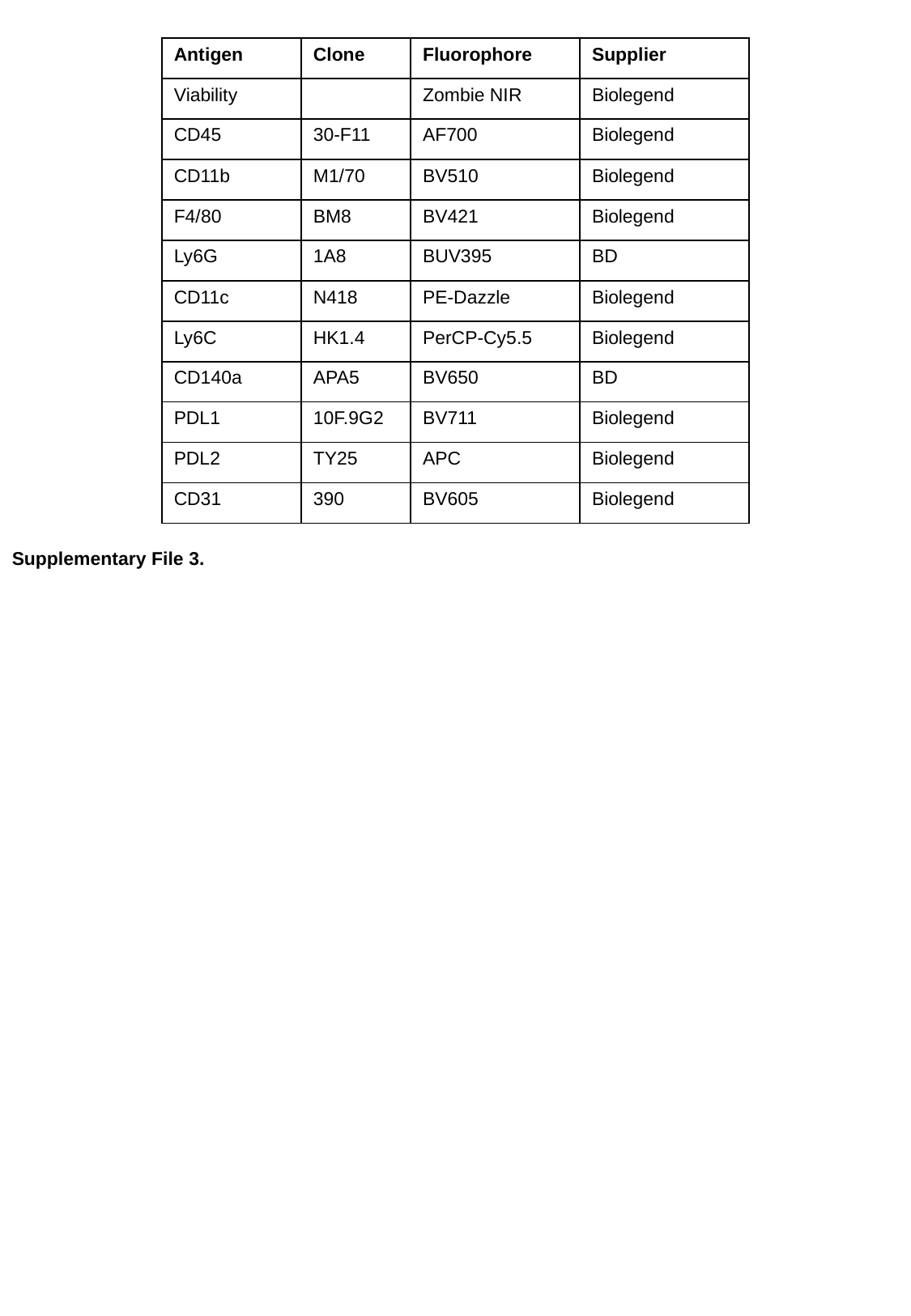

| Antigen | Clone | Fluorophore | Supplier |
| --- | --- | --- | --- |
| Viability | | Zombie NIR | Biolegend |
| CD45 | 30-F11 | AF700 | Biolegend |
| CD11b | M1/70 | BV510 | Biolegend |
| F4/80 | BM8 | BV421 | Biolegend |
| Ly6G | 1A8 | BUV395 | BD |
| CD11c | N418 | PE-Dazzle | Biolegend |
| Ly6C | HK1.4 | PerCP-Cy5.5 | Biolegend |
| CD140a | APA5 | BV650 | BD |
| PDL1 | 10F.9G2 | BV711 | Biolegend |
| PDL2 | TY25 | APC | Biolegend |
| CD31 | 390 | BV605 | Biolegend |
Supplementary File 3.
