# Supplementary material for: T-cell co-stimulation in combination with targeting FAK drives enhanced anti-tumor immunity
Source: eLife. 2020 Jan 21;9:e48092. doi: 10.7554/eLife.48092 (PMC6974352; doi:10.7554/eLife.48092)
Supplement: Supplementary file 4. [file elife-48092-supp4.pptx]

## Slide 1
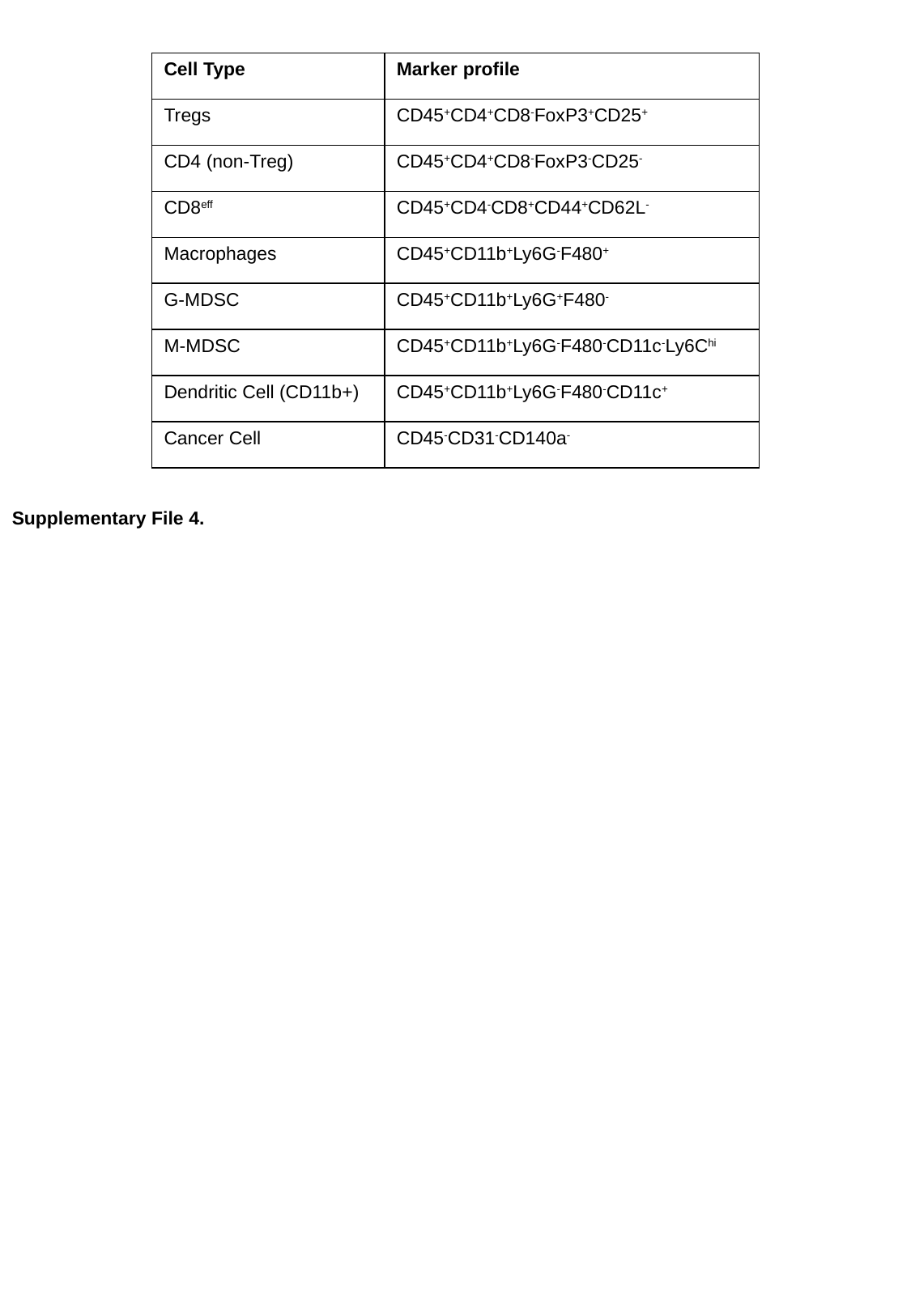

| Cell Type | Marker profile |
| --- | --- |
| Tregs | CD45+CD4+CD8-FoxP3+CD25+ |
| CD4 (non-Treg) | CD45+CD4+CD8-FoxP3-CD25- |
| CD8eff | CD45+CD4-CD8+CD44+CD62L- |
| Macrophages | CD45+CD11b+Ly6G-F480+ |
| G-MDSC | CD45+CD11b+Ly6G+F480- |
| M-MDSC | CD45+CD11b+Ly6G-F480-CD11c-Ly6Chi |
| Dendritic Cell (CD11b+) | CD45+CD11b+Ly6G-F480-CD11c+ |
| Cancer Cell | CD45-CD31-CD140a- |
Supplementary File 4.
