# Supplementary material for: T-cell co-stimulation in combination with targeting FAK drives enhanced anti-tumor immunity
Source: eLife. 2020 Jan 21;9:e48092. doi: 10.7554/eLife.48092 (PMC6974352; doi:10.7554/eLife.48092)
Supplement: Transparent reporting form [file elife-48092-transrepform.pdf]

eLIFE
1st Floor
24 Hills Road
Cambridge CB2 1JP, UK
P 01223 855340
W elifesciences.org
T @elife

eLife's transparent reporting form

We encourage authors to provide detailed information *within their submission* to facilitate the interpretation and replication of experiments. Authors can upload supporting documentation to indicate the use of appropriate reporting guidelines for health-related research (see EQUATOR Network), life science research (see the BioSharing Information Resource), or the ARRIVE guidelines for reporting work involving animal research. Where applicable, authors should refer to any relevant reporting standards documents in this form.

If you have any questions, please consult our Journal Policies and/or contact us: editorial@elifesciences.org.

Sample-size estimation

- You should state whether an appropriate sample size was computed when the study was being designed
- You should state the statistical method of sample size computation and any required assumptions
- If no explicit power analysis was used, you should describe how you decided what sample (replicate) size (number) to use

Please outline where this information can be found within the submission (e.g., sections or figure legends), or explain why this information doesn't apply to your submission:

An estimation of the group sizes required to achieve statistical significance for mouse studies was performed using power calculations based on a 1-Way ANOVA Pairwise 2-Sided Equality test. These calculations were performed using a power of 0.9, an error rate of 5%, and taking into account the number of pairwise comparisons for each experiment. Where possible, previously acquired data using the same tumor models was used for these calculations.

Replicates

- You should report how often each experiment was performed
- You should include a definition of biological versus technical replication
- The data obtained should be provided and sufficient information should be provided to indicate the number of independent biological and/or technical replicates
- If you encountered any outliers, you should describe how these were handled
- Criteria for exclusion/inclusion of data should be clearly stated
- High-throughput sequence data should be uploaded before submission, with a private link for reviewers provided (these are available from both GEO and ArrayExpress)

Please outline where this information can be found within the submission (e.g., sections or figure legends), or explain why this information doesn't apply to your submission:

The N number for each experiment is indicated in the figure legend.

Statistical reporting

- Statistical analysis methods should be described and justified
- Raw data should be presented in figures whenever informative to do so (typically when N per group is less than 10)
- For each experiment, you should identify the statistical tests used, exact values of N, definitions of center, methods of multiple test correction, and dispersion and precision measures (e.g., mean, median, SD, SEM, confidence intervals; and, for the major substantive results, a measure of effect size (e.g., Pearson's r, Cohen's d)
- Report exact p-values wherever possible alongside the summary statistics and 95% confidence intervals. These should be reported for all key questions and not only when the p-value is less than 0.05.

Please outline where this information can be found within the submission (e.g., sections or figure legends), or explain why this information doesn't apply to your submission:

The statistical tests used are detailed in the figure legends and the software used to perform the statistical tests is detailed in the methods section. N numbers are listed in the figure legends. P values are indicated on each graph and detailed in the figure legends.

(For large datasets, or papers with a very large number of statistical tests, you may upload a single table file with tests, Ns, etc., with reference to sections in the manuscript.)

Group allocation

- Indicate how samples were allocated into experimental groups (in the case of clinical studies, please specify allocation to treatment method); if randomization was used, please also state if restricted randomization was applied
- Indicate if masking was used during group allocation, data collection and/or data analysis

Please outline where this information can be found within the submission (e.g., sections or figure legends), or explain why this information doesn't apply to your submission:

For in vivo mouse experiments using only BI 853520, mice were randomly allocated into groups prior to the start of the experiment by an animal technician and oral dosing was performed by an animal technician who is independent of the research group. Tumor measurements were performed by two post-doctoral researchers and the agreed measurement recorded.

For in vivo mouse experiments using both BI 853520 and an immunotherapy, mice receiving either vehicle of BI 853520 were allocated randomly into groups prior to starting treatment with either isotype control antibody or immunotherapies. BI 853520 treatment was performed by an animal technician independent of the research group, and IP administration of antibodies was performed by an experienced post-doctoral researcher within the research group. Tumor measurements were performed by two post-doctoral researchers and the agreed measurement recorded.

Additional data files ("source data")

- We encourage you to upload relevant additional data files, such as numerical data that are represented as a graph in a figure, or as a summary table
- Where provided, these should be in the most useful format, and they can be uploaded as "Source data" files linked to a main figure or table
- Include model definition files including the full list of parameters used

- Include code used for data analysis (e.g., R, MatLab)
- Avoid stating that data files are "available upon request"

Please indicate the figures or tables for which source data files have been provided:

There are no large datasets associated with this study which are not already within the public domain. Nanostring gene expression analysis of SCC6.2 and SCC7.1 cells has been uploaded as a pdf.
